# Flow‐directed PCA for monitoring networks

**DOI:** 10.1002/env.2434

**Published:** 2016-12-21

**Authors:** K. Gallacher, C. Miller, E. M. Scott, R. Willows, L. Pope, J. Douglass

**Affiliations:** ^1^ School of Mathematics and StatisticsUniversity of GlasgowGlasgowU.K.; ^2^ Evidence DirectorateEnvironment AgencyU.K.

**Keywords:** connected monitoring networks, flow direction, PCA

## Abstract

Measurements recorded over monitoring networks often possess spatial and temporal correlation inducing redundancies in the information provided. For river water quality monitoring in particular, flow‐connected sites may likely provide similar information. This paper proposes a novel approach to principal components analysis to investigate reducing dimensionality for spatiotemporal flow‐connected network data in order to identify common spatiotemporal patterns. The method is illustrated using monthly observations of total oxidized nitrogen for the Trent catchment area in England. Common patterns are revealed that are hidden when the river network structure and temporal correlation are not accounted for. Such patterns provide valuable information for the design of future sampling strategies.

## 1 INTRODUCTION

Environmental monitoring networks are often designed with the aim of providing representative coverage of the spatial domain of interest and to provide a set of monitoring sites that can be used to identify variation and change in variables of interest over time. On a connected network, such as for a river, monitoring sites may share the same drainage catchment area and may be connected through river flow. Geochemical variation between drainage catchment areas induces spatial correlation in the water quality measurements that may be related to Euclidean distance and river discharge, with measurements also related over time. Redundancies are, therefore, introduced in the information provided by samples taken at such sites, and such correlation can mask identification of important patterns for determinands of interest within the network. This paper presents a novel statistical approach to identify such patterns after accounting for spatial network structure and temporal correlation.

The Environment Agency (EA) is the competent authority responsible for monitoring the environment in England, and one of their key responsibilities is to improve and maintain river water quality, applying standards defined by regulations implementing EU directives such as the Water Framework Directive, (European Parliament, 2000) and Nitrates Directive (European Parliament, 1991). Compliance with these directives is achieved in part by sampling the river networks in England over time and classifying the water quality of rivers and other waterbodies according to European standards. Identification of dominant spatial and temporal patterns in river network data can be used to identify areas where water quality has remained stable over time or to create groups of monitoring sites that exhibit similar temporal patterns. However, such patterns can be hidden in the presence of multiple layers of spatial and temporal correlation. Identification of common patterns could be used to improve the focus and design of water quality monitoring programs and inform future monitoring strategies, for example, by providing guidance to the appropriate position for placing automatic monitoring stations.

One approach to identify dominant spatial and temporal patterns is to use principal component analysis (PCA, Pearson, 1901; Hotelling, 1933), a dimension reduction technique where the aim is to replace *p* correlated variables with *k* < *p* uncorrelated variables, or principal components (PCs), that describe the main modes of variation in the data. The aim of this paper is to propose a novel development for PCA in order to improve identification of dominant spatial and temporal patterns in a flow‐connected network, with specific application to river water quality data. This paper proposes incorporating weight matrices in PCA methodology that reflect spatial and temporal autocorrelation and in particular proposes a method to construct a matrix of spatial weights to reflect the direction of water flow and strength of relationship between connected monitoring sites. The method is demonstrated using data collected from a densely monitored river network in England.

PCA is usually performed on multivariate data where columns are values of different variables and rows of a data matrix **X** are the sample units. For example, for water quality, rows could be locations on a river network, and column variables could be different water quality determinands. Some recent examples can be found in Wilbers, Becker, Sebesvari, and Renaud (2014); Shrestha, Kazama, and Nakamura (2008); Bengraïne and Marhaba (2003); and Petersen, Bertino, Callies, and Zorita (2001). Alternatively, data for a single variable might be recorded at several monitoring sites over time, and PCA can be used here to identify dominant spatial and temporal patterns. Specifically, Richman (1986) refers to two approaches: T‐ and S‐mode PCA, and the particular mode depends on whether the columns of **X** are time points (T‐mode) or monitoring sites (S‐mode).

T‐mode PCA aims to identify spatial patterns in the data and the associated time points at which these spatial patterns occur. The presence of more than one dominant spatial pattern suggests a change in the spatial pattern over time. Alternatively, the identification of a single dominant spatial pattern would suggest that the spatial distribution of the variable of interest has remained stable over time. Some recent examples include Zhang et al. (2012) (sea level pressure), Hidalgo‐Muñoz, Argüeso, Gámiz‐Fortis, Esteban‐Parra, and Esteban‐Parra (2011) (rainfall); and Barreira and Compagnucci (2011) (sea ice concentration). S‐mode PCA, also known as empirical orthogonal functions in the climatology literature, aims to estimate dominant temporal patterns in the data and to provide an indication of which sites exhibit similar temporal patterns. This is known as regionalization, and examples can be found in a variety of applications such as precipitation (Ehrendorfer, 1987; Neal & Phillips, 2009), surface wind (Jiménez et al. 2008), and streamflow (Kahya, Kalaycı, & Piechota, 2008). For river networks, if common temporal patterns can be identified, then this suggests potential redundancy in the monitoring network, and such information could be used to inform future sampling campaigns.

PCA utilizes correlation between variables to find structure in the data but does not explicitly make use of known structure, which in an environmental context could be spatial or temporal structure. An early example of adjusting PCA for known structure can be found in Gabriel and Zamir (1979) who develop a low‐rank approximation of matrices using weighted least squares for any choice of weights. Tamuz, Mazeh and Zucker (2005) develop a similar algorithm to remove known linear systematic effects from photometric light curves that is suitable for data with heterogeneous errors. Baldwin, Stephenson, and Jolliffe (2009) describe a general weighting scheme to account for known structure among variables using a diagonal weight matrix, although Allen, Grosenick, and Taylor (2014) discuss a generalized matrix decomposition where any symmetric weight matrix reflecting structure in the observations or variables can be incorporated into PCA using a weighted singular value decomposition (SVD).

PCA can be adjusted for spatial structure by combining PCA with Moran's I as in Wartenberg (1985); Thioulouse, Chessel, and Champely (1995); Jombart, Devillard, Dufour, and Pontier (2008); and Dray, Saïd, and Débias (2008). The aim in these papers is to find PCs that capture maximal variance and are spatially correlated. Alternatively, Harris, Brunsdon, and Charlton (2011); and Harris, Clarke, Juggins, Brunsdon, and Charlton (2015) describe geographically weighted PCA for areal unit data where PCA is adjusted for spatial heterogeneity rather than autocorrelation. Cheng et al. (2011) describe fuzzy masking PCA for image data where weights are used to constrain the analysis to focus on pixels with particular geology of interest. Frichot, Schoville, Bouchard, and François (2012) use weights based on the inverse of a spatial covariance matrix to uncover interesting spatial features previously masked by smooth transitions in space.

PCA can also be adjusted for temporal structure in the data such as in Ku, Storer, and Georgakis (1995) who develop dynamic PCA for statistical process control applications with temporally autocorrelated data by augmenting the data matrix with lagged variables. A different approach is taken by Stahlschmidt, Härdle, and Thome (2015) who adapt PCA for multivariate spatiotemporal data by applying PCA to a time averaged spatial covariance matrix.

The aim of this paper is to introduce a novel approach to PCA that accounts for direction dependent spatial autocorrelation and to apply it to a spatiotemporal dataset for a large river network catchment area in England. Specifically, the development and inclusion of an asymmetric matrix of spatial weights reflecting flow direction and strength of connectedness in a monitoring network is proposed as a methodological adaptation to PCA. The new PCA method will result in dimension reduction of a large dataset, in addition to enabling features within the data to be revealed that can be hidden by the presence of temporal and spatial correlation. The data are described in Section 2, followed by a description of the method in Section 3. An application of the method to data from the Trent catchment area is provided with discussion in Section 4. Section 5 contains a discussion of the new method with a conclusion provided in Section 6, and Section 7 gives details of tools developed using R statistical software that can be used to implement the methods introduced in this paper.

## 2 THE DATA

Data were provided by the EA for total oxidized nitrogen (TON), determined as the sum of nitrate (NO_3_) and nitrite (NO_2_), measured as mg/L, at 566 monitoring sites at approximately monthly intervals between 1990 and 2010. Nitrate and nitrite are bio‐available forms of the macro‐nutrient nitrogen. Excess nitrogen may lead to eutrophication (excessive algal growth) that can have many negative environmental impacts. Under European legislation, the highest acceptable concentration of nitrate in drinking water is 50 mg/L (European Parliament, 2000; 1991; 1975). Nitrate concentrations tend to be higher in areas designated as nitrate vulnerable zones where the main contributor to elevated nitrate levels is runoff from intensive agricultural practices, a form of diffuse source pollution (EEA, 2015). Nitrate levels are also affected by point source pollution such as sewage waste in areas of high urbanization and the spatial distribution of nitrates from such sources will be affected by river network topology.

**Figure 1:**
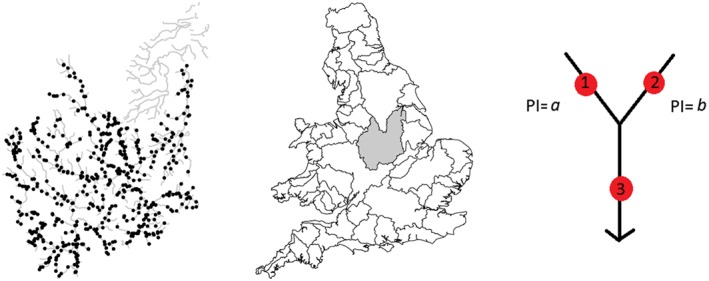
River network in Trent catchment area (gray lines) with 566 monitoring sites (black dots), (left). Location of Trent catchment area in England and Wales (middle). Diagram of simple river network with three monitoring sites and corresponding proportional influence (PI) values for upstream segments (right)

Observations of TON were collected at different sampling frequencies at each of 566 monitoring sites within the Trent catchment area, shown in Figure 1 (left and middle). A natural log transformation of TON observations was taken to stabilize the variance over time and across the network (Henderson, 2006); original values differed across the catchment area by two orders of magnitude. Two data sets were derived from the observations: data_*win*_ and data_*all*_. Data_*win*_ contain annual winter average log TON from 1995 till 2007 for 481 monitoring sites, which provide a time period and site combination with complete data. Data_*win*_ are of specific interest because levels of TON are typically higher in the winter and therefore more likely to exceed legal limits. Data_*all*_ contain observations for all 566 monitoring sites with monthly averaged log TON from 1990 to 2010. Because PCA requires a complete data set, missing values (approximately 30%) were imputed by implementing the method described in Josse and Husson (2012) using the R package missMDA. This imputation method makes use of correlation in the data to estimate missing values. Missing values are imputed by first applying standard PCA to the incomplete data where missing values are initially replaced with column means. Data are then reconstructed from a specified number of PC's, and PCA is repeated but with missing values replaced using estimates from the reconstructed data. This process is repeated until convergence, and the missing values in the original data are replaced with values estimated from the last PCA data reconstruction. This approach has been selected here to provide an imputation for the missing values that is within the same framework of the statistical modelling being introduced. The method assumes the data are missing at random or missing completely at random, which can be difficult to truly assess for environmental data. However, the aim here is to provide an estimate of PCs and assess the variability of the results due to missing values, rather than getting the best estimates of missing values. This imputation method is based on the EM algorithm, but is regularized to prevent overfitting, and allows estimation of the variability of the PCs axes due to missing values (Josse & Husson, 2012).

Seven PC's were used to impute missing values in data_*all*_, and this number was selected using k‐fold cross validation.

## 3 METHODOLOGY

### 3.1 Principal components analysis

PCA can be performed using SVD of the column mean centered *n* × *p* data matrix **X** such that

$$X=UDV^{⊤}$$

where **U** and **V** are the left and right singular vectors of **X** respectively and **D** is a diagonal matrix containing the singular values. Each PC is a linear combination of the original *p* variables, and the weights used to calculate the PC's are called loadings. In SVD, the loadings are found in the columns of **V**, and the PCs can be calculated as either **U** **D** or **XV**.

Assuming $\hat{X}=$ centered data reconstructed from *k* < *p* PCs then

(1) $$X=\hat{X}+\varepsilon_{rec}$$

where ***ε***_*rec*_, the reconstruction error, is the second term on the right side of (2), **V**_1:*k*_ indicates the first *k* columns of **V**, and **V**_*k* + 1:*p*_ indicates the last *p* − *k* columns. ***ε***_*rec*_ can also be calculated as the sum of squared differences between **X** and $\hat{X}$.

(2) $$X=XV_{1:k}V_{1:k}^{⊤}+XV_{k+1:p}V_{k+1:p}^{⊤}$$

### 3.2 Weighted PCA

Adjusting PCA for network structure and temporal autocorrelation can be achieved using appropriate row and column weights. A *p* × *p* column weight matrix **Ω** and *n* × *n* row weight matrix **Φ** can be constructed so that PCA is applied to

$$\tilde{X}=\Phi X\Omega =\tilde{U}\tilde{D}{\tilde{V}}^{⊤}.$$

However, the PCs and loadings are related to $\tilde{X}$ rather than **X**. Baldwin et al. (2009) and Allen et al. (2014) show that loadings and PCs can be calculated for **X** using a suitable back transformation, and call this the “general solution.” The back transformation can be defined by first considering (3) where the first term on the right is $\tilde{X}$ reconstructed using *k* PC's and the second term on the right is *ε*_*rec*_.

(3) $$\Phi X\Omega =\Phi X\Omega {\tilde{V}}_{1:k}{\tilde{V}}_{1:k}^{⊤}+\Phi X\Omega {\tilde{V}}_{k+1:p}{\tilde{V}}_{k+1:p}^{⊤}$$

The loadings and PCs can be backtransformed by pre‐multiplying the terms in (3) by **Φ** ^− 1^ and post‐multiplying by **Ω** ^− 1^ to give

$$X=X\Omega {\tilde{V}}_{1:k}{\tilde{V}}_{1:k}^{⊤}\Omega^{-1}+X\Omega {\tilde{V}}_{k+1:p}{\tilde{V}}_{k+1:p}^{⊤}\Omega^{-1}$$

The PCs are therefore $X\Omega \tilde{V}$, and the loadings are $\Omega^{-1⊤}\tilde{V}$.

### 3.3 Defining spatial weights for river networks

A matrix of spatial weights describing the flow direction and strength of relationship between monitoring sites on a river network can be incorporated into PCA methodology as either row or column weights, depending on the PCA mode of interest. Peterson and ver Hoef (2010) show how spatial weights reflecting the influence of upstream monitoring sites on downstream sites can be calculated based on discharge or proxy values for discharge such as watershed area (the area of land draining directly to a stream segment).

Figure 1 (right) shows a simple river network with three stream segments and three monitoring sites, as well as the proportional influence (PI) of two stream segments, joining at a confluence, on the downstream segment. PI∈[0, 1] and PI_*a*_ + PI_*b*_ = 1. Spatial weights are constructed by first calculating the product of all PI values between each stream segment and the stream segment whose most downstream point is the outlet. This product is called the additive function (AF), and monitoring sites are assigned the AF value for the segment on which they are located. Next, weights $\pi_{s_{u},s_{d}}$ reflecting the relative influence of an upstream monitoring site *s*_*u*_ on a downstream monitoring site *s*_*d*_, where *s*_*u*_ and *s*_*d*_ are connected by the flow direction of the river, can be calculated as $\pi_{s_{u},s_{d}}=\sqrt{\frac{\text{AF}(s_{u})}{\text{AF}(s_{d})}}$. AF(·) is the AF value for a monitoring site. Finally, a *p* × *p* matrix of spatial weights for *p* monitoring sites can be constructed by calculating $\pi_{s_{u},s_{d}}$ for all pairs of flow‐connected monitoring sites and the values entered into a matrix **S**, where columns are indexed by the upstream site ID and rows are indexed by the downstream site ID.

For weighted PCA, an asymmetric matrix of spatial weights can be calculated following the steps described in detail in Peterson and ver Hoef (2010), with the exception that the matrix is not forced to symmetry in the final step. For S‐mode PCA, **X** is arranged so that each column (variable) represents a monitoring site and each row (observation) represents the ordered time points. **S** must therefore be constructed so that rows represent upstream sites and columns represent downstream sites (i.e., water flows *from* rows *to* columns). This means that the diagonal elements of **XS** are a linear combination of the variance at each monitoring site and some proportion of variance from all flow‐connected upstream sites. The combination of an asymmetric weight matrix with this particular orientation of **X** and **S** preserves the flow direction of the river network. A symmetric weight matrix such as that used in Peterson, Theobald, and ver Hoef (2007) would result in the variance at a single site being a linear combination of the variances at all flow‐connected sites in both upstream and downstream directions, and it does not make physical sense that the variance at a monitoring site would be affected by the variance at sites downstream. For T‐mode PCA, **X** is arranged so that rows are monitoring sites and the matrix of spatial weights **S** must therefore be calculated such that water flows *from* columns *to* rows. This means that for T‐mode PCA the columns of the matrix of spatial weights represent upstream monitoring sites, and the rows are downstream sites. The orientation of **S** relative to **X** is crucial so that the direction of flow is correctly represented. See Peterson and ver Hoef (2010) for a simple diagram illustrating the process.

Once **S** has been calculated and is correctly oriented, PCA can be adjusted for known spatial structure using the inverse of the matrix square root such that **S**=**ss**, and therefore, the matrix of spatial weights is $s^{-\frac{1}{2}}$. The matrix square root can be calculated using the expm package (Goulet et al., 2014) in R. The use of the inverse square root of **S** to remove the effect of autocorrelation is in agreement with the discussions in Wartenberg (1985), Baldwin et al. (2009), and Allen et al. (2014). Frichot et al. (2012) also use inverse weights in a weighted factor analysis with the aim of uncovering interesting spatial features previously masked by spatial autocorrelation.

### 3.4 Defining temporal weights for river networks

A weight matrix **T** for temporal structure can be constructed such that **T** is an *n* × *n* symmetric matrix. In this work, **T** contains the elements *ρ* ^|*i* − *j*|^ where *ρ* is the strength of correlation between observations at time points 1,…,*n* − 1 and 2,…,*n* and *i* = 1,..,*n*; *j* = 1,…,*n*. This weight matrix therefore reflects temporal correlation with an AR(1) structure. There are many environmental examples where an AR(1) correlation structure is sufficient to model temporal correlation (Clement, Thas, Vanrolleghem, & Ottoy, 2006; or Andrés Houseman, 2005, for example). As with the matrix of spatial weights $S,T^{-\frac{1}{2}}$ is used to adjust PCA for temporal autocorrelation.

## 4 APPLICATION TO THE TRENT CATCHMENT AREA

This section will first describe the calculation of weights reflecting spatial and temporal structure in the Trent catchment area. This will be followed by the application of PCA adjusted for spatial and temporal structure in both T‐mode and S‐mode.

### 4.1 Spatial weights

The PI values were calculated for the Trent catchment area using area of land draining to a stream segment (km^2^). Drainage land area is a proxy for discharge, which assumes rainfall is relatively constant over the entire catchment area (Peterson et al., 2007; Peterson & ver Hoef, 2010), and values for each stream segment were obtained using the STARS toolkit (Peterson & Ver Hoef, 2014) in ArcGIS v9.3.

### 4.2 Temporal weights

The value for *ρ* in the Trent catchment area was estimated by fitting an additive model (Hastie & Tibshirani, 1990) to each of the 566 monitoring sites in the Trent catchment area separately to remove trend and seasonality from each time series, after which correlation between complete pairs of residuals was calculated for observations at time *t* and *t* − 1. The median correlation value from 566 sites was 0.27 with an interquartile range of 0.2–0.35, and so, *ρ* = 0.27 was used to construct **T**.

### 4.3 T‐mode PCA results

Firstly, it was of interest to investigate common spatial patterns over time. An unweighted T‐mode PCA (TPCA_*uw*_) was performed on mean centered annual winter data (data_*win*_), and this was followed by a row weighted T‐mode PCA (TPCA_*r*_) where PCA was adjusted for river network structure among observations using spatial weights as defined in Section 4.1. For TPCA_*uw*_, the first two PC's accounted for 89% and 3% of the variance in the data, respectively. The loadings for the first component are all of the same sign and of similar magnitude, and therefore, this PC represents the average spatial pattern over all years. Because the second PC accounted for only 3% of the variance in the data, it can be concluded that one PC is sufficient to describe the spatial pattern of winter log(TON) in the Trent catchment area. This means that the spatial pattern of winter TON has remained stable between 1995 and 2007. For TPCA_*r*_, the first two components accounted for 85% and 4%. Therefore, adjusting for spatial structure among the observations has led to a small reduction in the variance explained by the first PC. PCA uses correlation in the data to estimate PCs accounting for maximal variance. If data are independent, then each PC accounts for 100∗1/*p* *%* of total variance, whereas if data are completely correlated, then 1 PC will account for 100% of the variance. Adjusting PCA for spatial correlation using inverse weights means that some of the correlation is removed, and hence, the first weighted PC accounts for a smaller percentage of the variance than the first unweighted PC. This can help tease out patterns, particularly where data are highly correlated in space (Frichot et al., 2012).

**Figure 2:**
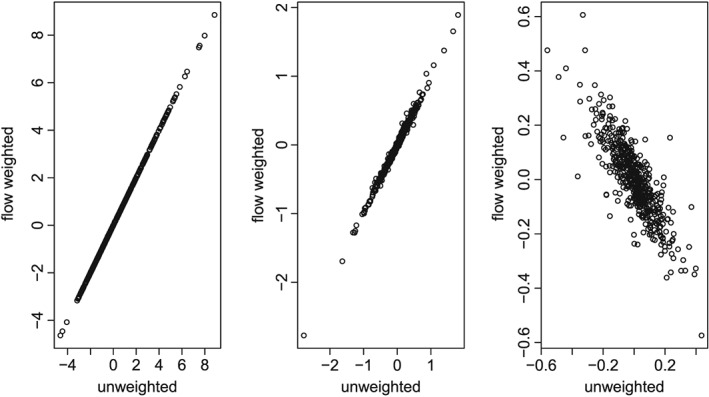
Principal component scores for TPCA_*uw*_ (unweighted PCA) and TPCA_*r*_ (spatially weighted PCA), for PCs 1 (left), 2 (middle), and 13 (right). Note: plots are on different scales

Figure 2 shows that differences in the principal components between TPCA_*uw*_ and TPCA_*r*_ are most evident for the PCs that explain the smallest proportion of the variance in the data, corresponding to the noise structure. This makes sense because the row weights reflect spatial network correlation in what remains after removing trend, and so the biggest differences between TPCA_*uw*_ and TPCA_*r*_ are found beyond the first few PCs. Frichot et al. (2012) also noticed that differences between standard PCA and PCA adjusted for spatial correlation among observations using inverse weights were more prominent in the second and third PCs rather than the first PC. Additional plots of the results can be found in the Supporting Information.

The results of incorporating the spatial weights here are as expected but are small for this simple example of complete annual winter data. T‐mode PCA both with and without the adjustment for spatial correlation illustrated that the spatial pattern of winter log(TON) has remained stable over time. It appears that the highest levels of log(TON) were found in the East and South‐east of the river network, and log(TON) has remained low and stable over time in the North‐west. Such results could potentially be used to concentrate future monitoring in the areas where log(TON) is highest. The areas with high levels of log(TON) have remained stable over time. However, these areas must still be monitored to ensure that the 50 mg/L limit is not exceeded. Maps of the PCs from TPCA_*uw*_ and TPCA_*r*_ showing areas where log(TON) has remained low/high over time can be found in the Supporting Information.

### 4.4 S‐mode PCA results

The simple T‐mode PCA example was provided to illustrate the methods. However, S‐mode PCA will now be applied on the full spatiotemporal data, data_*all*_, after missing values have been imputed. An unweighted S‐mode PCA (SPCA_*uw*_) was applied to monthly observations data_*all*_ for the 566 monitoring sites in the Trent catchment area, to investigate sites with common temporal patterns. Following this, a column weighted PCA (SPCA_*c*_) was applied to adjust for known spatial network structure among sites and a row and column weighted PCA (SPCA_*rc*_) was also applied to additionally adjust PCA for temporal structure among the observations.

Table 1 gives the results from SPCA_*uw*_, SPCA_*c*_, and SPCA_*rc*_. For SPCA_*uw*_, the first component explains 42% of the variance in the data. Adjusting for spatial structure means this reduces to 38%, and adjusting for both spatial and temporal structure means that the first PC accounts for 31% of the variance in the data. The first three components (var_3_ in Table 1) for SPCA_*uw*_, SPCA_*c*_, and SPCA_*rc*_ account for 57%, 52%, and 43% of the variance, respectively.

**Table 1:** Results from SPCA_*uw*_(unweighted PCA), SPCA_*c*_(spatial weights), and SPCA_*rc*_(spatial and temporal weights)

PCA	PC1 (%)	PC2 (%)	PC3 (%)	var_3_ (%)	*k*	var_*k*_ (%)	***ε***_*rec*_
SPCA_*uw*_	42	9	6	57	8	70.8	9,069
SPCA_*c*_	38	9	5	52	12	70.5	8,354
SPCA_*rc*_	31	7	5	43	23	70.1	6,910

PC1‐3 contains % variability explained for each of the PCs, respectively; var_3_ is the % variability explained by the first three principal components; *k* is the number of principal components retained to explain at least 70% of the variance of the data; var_*k*_ is the amount of variance explained by *k* principal components; ***ε***_*rec*_ is the reconstruction error from *k* principal components.

**Figure 3:**
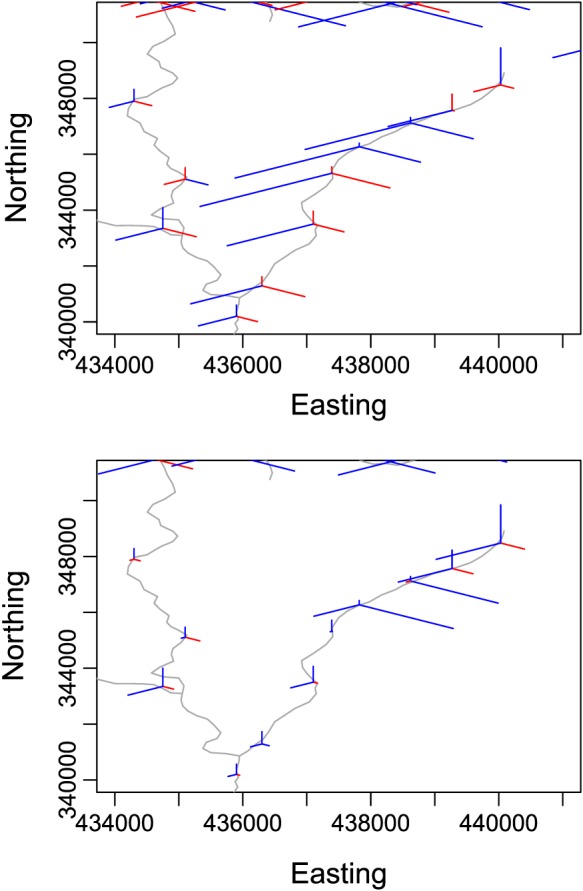
Glyph plots with loadings for the first three principal components from SPCA_*uw*_ (top – no weights) and SPCA_*rc*_ (bottom – weights for discharge and time) for a zoomed in section of the network. (Red indicates negative values, and blue indicates positive values, in online version). Length of line indicates relative magnitude of loading. Starting at the 12 o'clock position, the length of the line reflects the magnitude of the loading for the first PC, and moving clockwise, the other lines represent the loadings for subsequent PCs

In S‐mode PCA, maps of the loadings can be used to show which monitoring sites exhibit similar temporal patterns (Ehrendorfer, 1987) where two sites are “similar” if their loadings are of the same sign and similar magnitude. For a single‐monitoring site, a high loading (of either sign) means that the temporal pattern described by the PC with which the loading is related is found at that site. Figure 3 displays a zoomed in portion of glyph maps (Harris et al., 2011) of the loadings for the first three PCs from SPCA_*uw*_ (top) and SPCA_*rc*_ (bottom) for a small section of the monitoring sites on the Trent network. The top panel displays results from standard PCA (SPCA_*uw*_) and suggests that, moving North to South along the east branch of the displayed network, the seven southernmost monitoring sites are similar in relation to the third PC. However, the bottom panel displays results for SPCA corrected for both spatial river network structure and temporal correlation and shows that the four southernmost sites on the east branch are different from the northern sites. The variance along this stretch of river appears to be largely driven by the fifth and sixth sites from the bottom, and the fourth site from the bottom has small loadings for all three PCs and therefore contributes little to the variance on this river. The upper panel (standard PCA) suggested that all eight sites contribute equally to the variance along the river, and so, adjusting for spatial and temporal correlation means that the most and least influential monitoring sites can be identified after the masking effect of autocorrelation has been removed. Further plots of the results can be found in the Supporting Information.

In order to explain at least 70% of the variance (var_*k*_ in Table 1), SPCA_*uw*_ requires eight components, and *k* increases to 12 for SPCA_*c*_ and 23 for SPCA_*rc*_. Although a larger number of PCs are required after accounting for flow‐connectedness and temporal correlation here, the reconstruction error in (1), calculated using *k* retained PCs (***ε***_*rec*_ in Table 1), decreases by 24% when PCA is adjusted for both spatial and temporal structure for the same % of variability explained, and it is clear from Figure 3 that sites contributing to explaining the temporal patterns for each PC can be more easily identifed.

S‐mode PCA has shown that at least eight PC's are required to explain a large ( > 70*%*) amount of the variance in the data. The temporal pattern of monthly log(TON) over a 21‐year period appears to have been highly variable because the first PC represented less than half the variance in the data, for weighted and unweighted S‐mode PCA. Adjusting S‐mode PCA for spatial and temporal autocorrelation highlighted that more than 70% of the variance in the data could be explained by 23 PCs, and so, the temporal evolution of log(TON) across the whole monitoring network of 566 monitoring sites can be understood using only 23 temporal patterns, rather than individually inspecting 566 separate temporal patterns. Adjusting for spatial and temporal autocorrelation meant that the most (and least) influential sites could be more clearly identified as these were previously masked by smooth transitions along the river network.

## 5 DISCUSSION

Flow‐directed PCA that accounts for temporal correlation can efficiently identify spatiotemporal patterns across a network removing the masking effects of multiple layers of correlation. Adjusting T‐ and S‐mode PCA for spatial and/or temporal autocorrelation meant that the amount of variance explained by the first PC decreased. This is because the variance across space at each time point and the variance over time at each monitoring site is not independent of other time points/monitoring sites. For example, in S‐mode PCA, the diagonal of the covariance matrix represents the variance over time at each monitoring site, but each diagonal element will include the variance contributions from monitoring sites further upstream. Using inverse weights based on autocorrelation means that variance contributions from upstream sites are removed, and in flow‐weighted PCA, the reduced amount of total variance explained by the first PC can be thought of as the amount of variance explained once dependencies based on the river network structure are removed. In the case of river networks, this means that correlation related to land use or other Euclidean distance‐based relationships becomes the focus of the analysis, and it is therefore possible to tease apart different forms of spatial relationships among monitoring sites on a river network.

The strength of spatial and temporal autocorrelation in the data affects the additional insight that can be gained by applying spatiotemporally weighted PCA. Allen et al. (2014) found that in the case of high frequency data with strong temporal correlation, adjusting PCA for temporal correlation resulted in the identification of temporal patterns that were clearly separated from noise. Temporal correlation was weak (*ρ* = 0.27) in the example presented here, and so, it is to be expected that the temporal patterns estimated using S‐mode PCA were quite similar before and after adjusting for temporal correlation. However, adjusting for spatial network structure in the Trent example highlighted the most influential monitoring sites in the network, after applying spatially weighted S‐mode PCA, which were masked by the effects of spatial autocorrelation when standard PCA was applied. Frichot et al. (2012) also showed that it is possible to identify features previously masked by spatial autocorrelation using the inverse of weights representing spatial autocorrelation. The effect of incorporating the flow‐connected weights will depend on the contribution of the measured determinand that a monitoring site receives from upstream and that which drains to the site from the immediate surrounding waterbody.

Currently, regulatory agencies are investigating where efficiencies can be made in the monitoring budgets for river networks. In the Trent example, adjusting PCA for spatial and temporal autocorrelation, in particular SPCA_*rc*_, more clearly identified the most and least influential sites in the network and such knowledge, could be used to better focus the monitoring in space. The methods proposed in this paper can be generalized to account for different temporal correlation structures and to define spatial weights using alternative determinands (if available) such as observed (or interpolated) rainfall or discharge.

## 6 CONCLUSION

Flow‐directed PCA is a novel approach proposed here to investigate reducing dimensionality of spatiotemporal network data and identify common patterns. A novel adaptation of the T‐ and S‐mode PCA methodology was proposed to incorporate an asymmetric weight matrix reflecting spatial structure in the data, where spatial structure reflects flow direction and strength of connectedness in the monitoring network. The orientation of the asymmetric weight matrix in relation to the data matrix is crucial so that direction dependent relationships between monitoring sites are correctly represented. This methodology improves identification of dominant temporal patterns and interesting spatial features previously masked by autocorrelation. Improving the estimation of common temporal patterns in the data can provide regulatory agencies with evidence to inform future sampling strategies.

Although this work is motivated by an application to river networks, it is expected that the method developed here could be applied to data from any direction dependent monitored network.

## 7 SOFTWARE

R code (R Core Team, 2016) to implement the analyses in this paper can be accessed at https://doi.org/10.5525/gla.researchdata.277. A demonstration dataset is available with this package.

## Supporting information

Supporting info item
