# Supplementary material for: Flow‐directed PCA for monitoring networks
Source: Environmetrics. 2016 Dec 21;28(2):e2434. doi: 10.1002/env.2434 (PMC5347935; doi:10.1002/env.2434)
Supplement: Supplementary file 1 — Supporting info item [file ENV-28-na-s001.pdf]

Flow-directed PCA for monitoring networks: Supplementary material

1 T-mode results

Figure 1 shows the biplot for the first two TPCA$_{uw}$ (unweighted) principal components (left). Sites (blue numbers) in the same quadrant as arrow heads have high log(TON) in the years indicated by the red arrow labels so sites in the top and bottom right quadrants have low log(TON) in all years while sites in the top and bottom left quadrants have high log(TON) in all years. The arrows representing the loadings show that the first component is an average of the spatial patterns for all years while the second component is a contrast between 1995-1999 and 2000-2007. The right plot in Figure 1 shows the biplot for the first two TPCA$_r$ (spatially weighted) principal components. The years 2000-2007 are a little more closely grouped together than for the TPCA$_{uw}$ and 1995 is more similar to 1996/1997 than 1998/1999 for the TPCA$_{uw}$.

Figure 2 shows the dominant spatial pattern for winter log(TON) 1995-2007 for both TPCA$_{uw}$ and TPCA$_r$. Since this pattern accounts for over 80% of the variability in the spatial pattern over time it can be concluded that the spatial pattern has remained stable, strengthening the argument for reducing the number of monitoring sites so that sampling might be concentrated in areas where log(TON) is highest and more likely to exceed the upper limit set by the Water Framework Directive. Areas exhibiting low or high values for PC1 correspond to land use in the Trent Catchment area with low values found in urban areas and high values in agricultural areas (Gallacher, 2016).

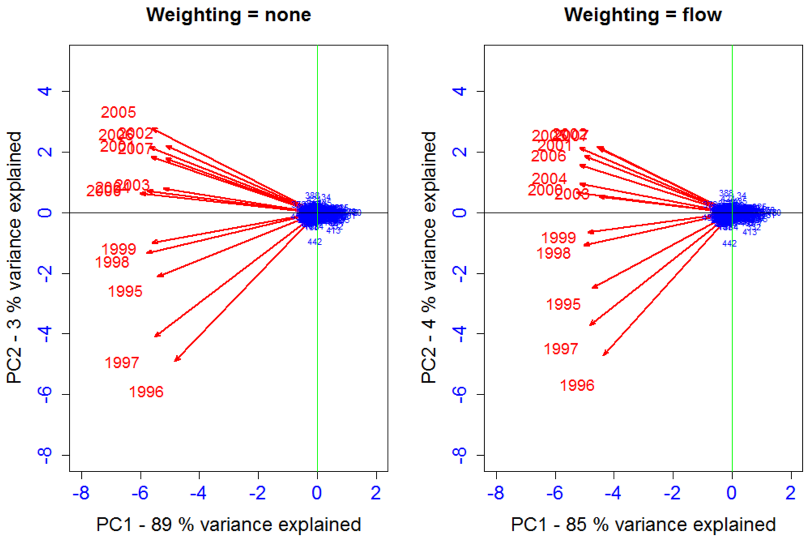

Figure 1: Biplots for the TPCA$_{uw}$, unweighted (left) and TPCA$_r$, spatial weights (right). Red arrows indicate sign and magnitude of loadings. Blue numbers are principal components/scores for monitoring sites.

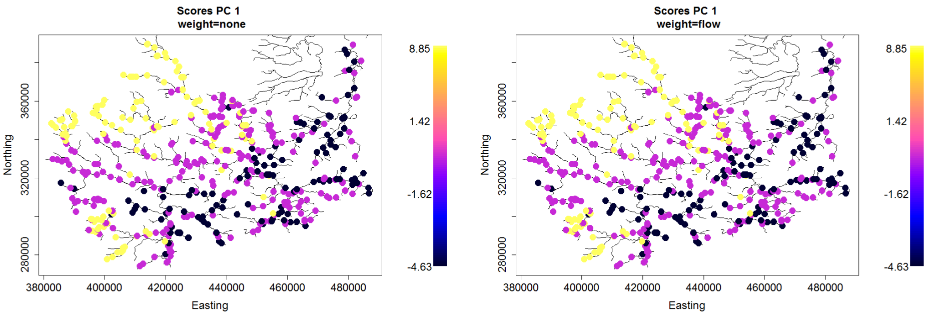

Figure 2: Scores for PC1 for TPCA$_{uw}$, unweighted (left) and TPCA$_r$, spatial weights (right). Black dots are scores in the lower quartile and yellow dots are scores in the upper quartile.

2 S-mode results

Time plots of the principal components for the first three PC's are in Figure 3. The first PC from SPCA$_{uw}$ (unweighted) estimates a temporal pattern with greater variability in early years compared to later years and a decrease in log(TON) from around 1999. The second principal component shows greater variability in the early part of the time period under consideration and a small increase in log(TON) in later years while the third component shows a peak in log(TON) around 1997 followed by a shallow decline in log(TON). The temporal pattern of log(TON) is slightly less variable around 1996 for SPCA$_c$ (spatial weights) than for the first unweighted principal component and the second principal component for SPCA$_c$ highlights greater variability in the early years with an increase in log(TON) between 1990 and 2000. The third principal component for SPCA$_c$ shows a spike in log(TON) around 1997, similar to the third unweighted principal component. For SPCA$_{rc}$ shown on the right of Figure 3 the first principal component is less variable in the first half of the time period than for SPCA$_{uw}$ but the third SPCA$_{rc}$ (spatial and temporal weights) principal component seems to capture the extra variability.

Figure 4 shows mean-centred average log(TON) for monthly observations across 566 sites $\pm$ a small multiple of the PC's in Figure 3 to show the effect of the temporal patterns estimated using SPCA$_{uw}$, SPCA$_c$ and SPCA$_{rc}$ on the mean temporal pattern, borrowing an idea from functional PCA (Ramsay and Silverman, 2006). The plots are limited to the first half of the time period to highlight differences between the weighted and unweighted analyses more clearly. The plots in the left panel show that there is almost no difference between the first principal component for SPCA$_{uw}$, SPCA$_c$ and SPCA$_{rc}$

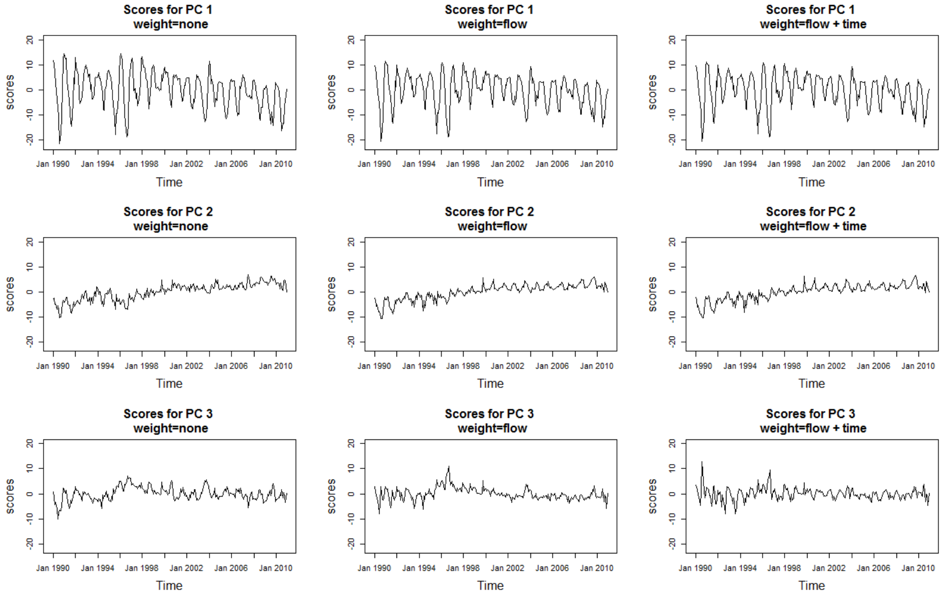

Figure 3: Scores plots for first three principal components from PCA_{uw} (unweighted), PCA_c (spatial weights) and PCA_{rc} (spatial and temporal weights).

except for the variability around the peak at January 1996 where SPCA_{uw} estimates greater variability around that point than the weighted analyses. For the second PC, log(TON) is less variable around January 1995 for SPCA_{uw} and more variable around January 1996 than SPCA_c and SPCA_{rc}. Greater differences are seen between the three analyses in the third principal component, in particular the variability around the troughs in January 1990, 1991 and 1992 is quite different between SPCA_{uw}, SPCA_c and SPCA_{rc}. S-mode PCA has shown that PC1 reflects variation around the mean such that the periodic signal is dampened down, indicated by the crossover pattern when a small amount of the principal component is added to or subtracted from the mean temporal pattern. PC2 reflects a shift in mean value while PC3 for SPCA_{uw} and SPCA_c correspond to a small time lag, indicated by the crossover pattern of adding or subtracting a small amount of the PC to the mean pattern and variability occurs around the slope rather than the turning points.

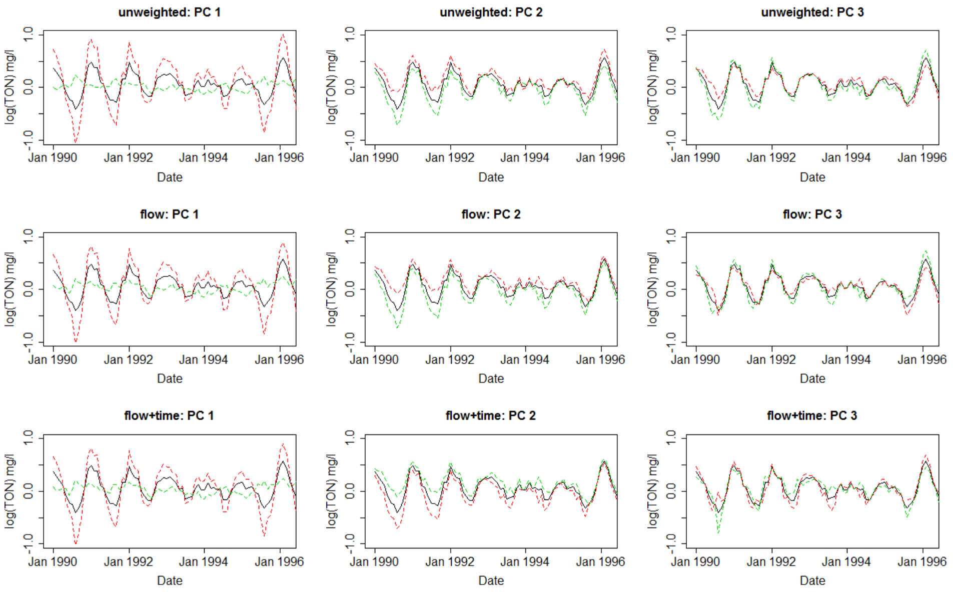

Figure 4: Mean-centered average log(TON) for 566 monitoring sites (solid black line) with + (green dashed line) and - (red dashed line) a small proportion of the principal component scores from SPCA$_{uw}$, SPCA$_c$ and SPCA$_{rc}$.

References

Gallacher, K. (2016). *Using river network structure to improve estimation of common temporal patterns.* Thesis, University of Glasgow, Glasgow, UK.

Ramsay, J. and B. Silverman (2006). *Functional Data Analysis.* Springer, New York.
